# Interplay Between Sociodemographic Variables, Physical Activity, Sleep, Dietary Habits, and Immune Health Status: A Cross-Sectional Study From Saudi Arabia's Western Province

**DOI:** 10.7759/cureus.33211

**Published:** 2023-01-01

**Authors:** Azzah S Alharbi, Sarah A Altwaim, Ali S Alharbi, Salhah Alsulami

**Affiliations:** 1 Medical Microbiology and Parasitology, King Abdulaziz University Faculty of Medicine, Jeddah, SAU; 2 Special Infectious Agents Unit, King Fahd Medical Research Center, Jeddah, SAU; 3 Medical Microbiology, Faculty of Medicine, Imam Abdulrahman Bin Faisal University, Jeddah, SAU; 4 Faculty of Medicine in Rabigh, King Abdulaziz University, Jeddah, SAU

**Keywords:** dietary habits, physical activity, sleep, smoking, obesity, lifestyle factors, immune status questionnaire

## Abstract

Background: In addition to developing effective therapeutic approaches, the maintenance of health also constitutes lifestyle and behavioral aspects related to being more resilient in the event of future illness. Reduced immune health has been linked to reports of more frequent and severe infections as well as a variety of non-communicable diseases, both of which may eventually place a significant burden on the healthcare system. Several lifestyles and behaviors can influence immune health, both positively and negatively. Accordingly, this study aimed to evaluate the immune health status and investigate its relationship with widely practiced lifestyle behaviors that are thought to affect immunological functioning.

Design and method: Saudi Arabian citizens and international residents of the Western Province were invited to participate in this cross-sectional web-based survey through an online advertisement. The integrated questionnaire on lifestyle (Arab Teens Lifestyle Study) and immune health status (Immune Status Questionnaire (ISQ)) was completed in November 2022 by 1230 participants. Descriptive analysis, Mann-Whitney U test, chi-square, or Fisher’s exact test was utilized to investigate the relationships between study variables and immune health status groups. Spearman's or Pearson’s correlation coefficients were used to determine correlations between the overall ISQ scores and study variables.

Results: Of the 925 study participants, 34.7% scored below 6 on the ISQ. Of the respondents, 50% had a body mass index of 25 or higher, and 46.3% reported sleeping less than four hours each night. Of the participants, 62-82% did not engage in any form of physical activity. The associations between the ISQ score and weight (p = 0.006), total sleep time per night (p = 0.001), duration of household activities (p < 0.001), and smoking status (p = 0.001) were statistically significant.

Conclusions: According to the data presented here, reduced immune health as measured by ISQ < 6 was prevalent among residents of Saudi Arabia's Western Province and correlated significantly with obesity, sleep duration, and smoking status. Various measures to mitigate the negative impact of an unhealthy lifestyle on public health and to reverse the observed poor immune health and their economic consequences are highly required.

## Introduction

Maintenance of health, prevention of and recovery from disease, as well as one’s general quality of life are all dependent on a resilient immune system that functions properly to eliminate harmful agents and return to a steady state after healing has occurred [1]. Beyond its effects on infection acquisition, poor immune health is related to numerous diseases, including non-communicable diseases such as cardiovascular illnesses, malignancies, and diabetes [2].

In addition to genetic factors, multiple lifestyle and psychological factors may influence immune function [3]. Mentally, physically, and nutritionally abnormal conditions have direct effects on human health, which are mediated in part by the immune system [4]. Aging is a frequent, non-modifiable factor associated with a decreased immune response to viral and bacterial infections, poor vaccination response, and increased risk of autoimmune disorders [5-7]. Recently, it was discovered that controllable factors such as sleep, stress, nutrition, smoking, and exercise can alter immunity [8]. During the recent novel coronavirus disease 2019 (COVID-19) pandemic in Saudi Arabia, stress, depression, and anxiety were observed to reduce immune function [9]. Obesity was considered an independent risk factor for disease severity during the COVID-19 pandemic [10]. A deviation from normal weight (BMI > 24.9) is associated with lower immune health status [11]. Obesity and its association with immunological weakness and increased susceptibility to infection have recently been established on a global scale. Furthermore, there is a significant relationship between healthy diet adherence and immune health [12]. The healthy diet score (HDS) and perceived immune fitness are significantly correlated [12]. The amount of fruit and vegetables consumed has an inverse relationship with inflammatory markers such as homocysteine and C-reactive protein (CRP) [13]. Micronutrients, including vitamins A, D, C, E, B6, and B12, folate, zinc, iron, copper, and selenium, are essential in every stage of the immune response [14]. Aerobic exercise has been reported to reduce inflammatory responses in animal models [15]. Recent in vitro and in vivo studies have significantly increased the amount of evidence of the role of sleep in immunological health status [16]. Short sleep duration and poor sleep quality are linked to shorter lymphocyte telomere length in obese males and females [17]. A previous study found that sleep quality, sleep apnea, and insomnia disorders were considerably greater among Dutch students with reduced immunity [18]. Sleeping for less than five hours increases the risk of various infections [19].

The prevalence of obesity in Saudi Arabia, according to a recent survey, is 24.7% [20], which is higher than the global average. A comprehensive Saudi study in multiple cities revealed that 34.4% of participants did not adhere to a healthy diet [21]. A limited minority of Saudis followed dietary recommendations, particularly in the consumption of fruits and vegetables, dairy products, nuts, and fish [22]. Moreover, the prevalence of smoking in Saudi society ranges from 12.2% to 34.4% and is viewed as a major concern [23]. The bulk of these lifestyle choices that affect one's immune health are under the control of the person and are quite easy to change [24]. To the best of our knowledge, no public health data from Saudi Arabia investigating immune health status (IS) and its relation to commonly adopted lifestyles have been published. As a result, we conducted a cross-sectional study to assess immune health status and examine its potential links with various lifestyle habits among Western Province residents.

## Materials and methods

Study design and participants

This study was cross-sectional and exploratory in nature. The Research Ethics Committee at King Abdulaziz University (KAU) approved the study protocol (reference number: 495-22), and it was conducted in conformity with the Helsinki Declaration. The last author's institutional account was used to create the questionnaire in Google Forms. It was made available online by public link and the link was shared via several social media platforms (i.e., Twitter, Facebook, WhatsApp, and Instagram). Saudi citizens and residents (>18 years old) from the Western Province were asked to fill in the questionnaire. The participants were encouraged to distribute the survey link to their networks using social media or instant messaging services within the same time frame. The survey was voluntary, and all responses were kept confidential. At the start of the questionnaire, the study's goals and rationale for data collection were stated explicitly. Participants’ consent was also recorded online before any responses were provided. The questionnaire was available only to those who agreed to participate. There was a block of multiple responses per participant. The study did not include participants who had a history of medical conditions or who were taking any kind of prescription medication. The survey was distributed on November 8, 2022. The intended sample size was determined after a month of data collection.

Questionnaire content

Sociodemographic information was collected on participants’ age, gender, self-reported anthropometric measurements (height and weight), marital status, earnings, citizenship, and city of residence in Saudi Arabia's Western Province. Using the following formula, BMI was calculated: body weight (kg) divided by height (m2). The Arab Teen Lifestyle Study (ATLS) was used to gather lifestyle information [25]. This questionnaire has already been demonstrated to be valid and reliable [26]. The questionnaire gathered data on physical activity, sedentary behavior, dietary habits, sleep duration, and smoking status. Participants’ immunological health over the previous 12 months was assessed using the Immune Status Questionnaire (ISQ) [11,27,28]. The ISQ is a seven-item measure with a five-point Likert scale ranging from never, occasionally, regularly, often, and always for rating the frequency of sudden high fever, diarrhea, headache, skin problems, muscle and joint soreness, common cold, and cough. The overall ISQ score ranged from 0 to 10, with a cut-off value of 6 indicating a decreased level of immunological fitness during the previous year. Numerous studies have demonstrated the validity and reliability of the ISQ [28]. On a scale from 0 (worst) to 10 (best), single-item questions were also used to subjectively assess current perceived immunological and general health states [2,11,18,28]. The questionnaire was made available in both English and Arabic to make it easier for those who could speak either language to participate. All study participants were also asked if they had changed their usual lifestyle behaviors in the last 12 months. It took approximately 10 minutes to complete the questionnaire.

Statistical analysis

The survey data were stored in Microsoft Excel (Microsoft Corporation, Redmond, WA) and analyzed using IBM SPSS Statistics version 20 software (IBM Corp., Armonk, NY). Categorical data are presented as frequencies and percentages, and continuous variables are presented using descriptive statistics (e.g., mean, standard deviation, median, and interquartile range). The Shapiro-Wilk test was used to determine whether each variable had a normal distribution, which would show a non-normal distribution. The participants were divided into two groups based on their ISQ scores: those with reduced IS (ISQ < 6) and those with normal IS (ISQ ≥ 6). To investigate the relationships between sociodemographic and lifestyle factors and IS groups, the Mann-Whitney U, chi-square, or Fisher’s exact test was utilized. Spearman's or Pearson’s correlation coefficients were used to determine correlations between the overall ISQ scores and study variables. All statistical tests were two-tailed. Statistical significance was indicated by a p-value < 0.05.

## Results

A total of 1230 responses were collected. Of these, 305 were excluded. A total of 184 responses from participants who had a medical condition or were taking medication were excluded. Due to the participants' age being under 18 years or their inconsistent responses, another 121 responses were eliminated. Data from the remaining 925 respondents were analyzed. An overview of the sociodemographic and lifestyle variables of the participants and other study outcomes is shown in Tables 1, 2. The vast majority of participants (76.7%) were between the ages of 18 and 39 years, with only 2.4% being 60 years or older. The male proportion in the sample was slightly higher than that of women (51.6% vs. 48.4%). The majority of participants (53.6%) were from Jeddah, the largest city in Saudi Arabia's Western Province. Only 12% were foreigners living in Saudi Arabia. In total, 30.2% of participants smoked, 50% had a BMI greater than 25, 46.3% slept for less than four hours per night, and 58.8% watched television and used computers for three hours or more per day. The participants were split into two groups based on their ISQ scores, and it was found that 34.7% of them had decreased IS (ISQ < 6), with a mean and standard deviation of 3.41 ± 1.7, and that 65.83% of them had normal IS (ISQ ≥ 6), with a mean and standard deviation of 8.5 ± 1.4. There were statistically significant differences between the IS groups in terms of gender, nationality, and residential location (P < 0.05). Subjects with decreased IS were significantly shorter (1.62 ± 0.15 vs. 1.66 ± 0.15; P = 0.001), had a higher weight (69 ± 22 vs. 65 ± 22; P = 0.006), and had poorer sleep (sleeping hours/night: 3 ± 3 vs. 4 ± 2; P = 0.001) than subjects who reported a normal IS. Statistically significant differences were observed. Table 2 shows that smokers had a higher prevalence of reduced IS (69.5%) compared to non-smokers (29.5%; P = 0.001).

**Table 1:** Sociodemographic characteristics of the enrolled study subjects grouped by ISQ scores Note: Data presented as N: number (%). Test used = chi-square or Fischer's exact test. Significant differences (p < 0.05) are indicated by *. ISQ: Immune Status Questionnaire.

Variables	Total (N = 925)	ISQ scores below cutoff value (N = 316) (34.17)	ISQ scores above cutoff value (N = 609) (65.83)	P
	N (%)	N (%)	N (%)	
Age (years)				0.915
18-39	710 (76.7)	245 (77.5)	465 (76.4)	
40-59	193 (20.9)	64 (20.3)	129 (21.2)	
≥60	22 (2.4)	7 (2.2)	15 (2.5)	
Gender				0.001*
Male	477 (51.6)	194 (61.4)	283 (46.5)	
Female	448 (48.4)	122 (38.6)	326 (53.5)	
Nationality				0.006*
Saudi	814 (88)	290 (91.8)	524 (86.0)	
Non-Saudi	111 (12)	26 (8.2)	85 (14.0)	
Social status				0.802
Single	513 (55.5)	179 (56.6)	334 (54.8)	
Married	373 (40.3)	122 (38.6)	251 (41.2)	
Divorced	27 (3)	11 (3.5)	16 (2.6)	
Widowed	12 (1.2)	4 (1.3)	4 (1.3)	
Monthly income in riyals				0.230
Less than 5,000	440 (47.6)	154 (48.7)	286 (47.0)	
5,000-10,000	194 (21)	75 (23.7)	119 (19.5)	
10,000-15,000	141 (15.2)	45 (14.2)	96 (15.8)	
15,000-20,000	76 (8.2)	24 (7.6)	52 (8.5)	
>20,000	74 (8)	18 (5.7)	56 (9.2)	
Residential location				0.001*
Jeddah	496 (53.6)	145 (45.9)	351 (57.6)	
Rabigh	115 (12.4)	60 (19.0)	55 (9.0)	
Others	314 (33.9)	111 (35.1)	203 (33.3)	
Living with				0.584
Family	807 (87.2)	272 (86.1)	535 (87.8)	
Separate	83 (9)	32 (10.1)	51 (8.4)	
Friend	22 (2.4)	9 (2.8)	13 (2.1)	
Other	13 (1.4)	3 (0.9)	10 (1.6)	

**Table 2:** Lifestyle-related variables of the enrolled study subjects grouped by ISQ scores Note: Data reported as median (interquartile range) or number (%). Test used = Mann-Whitney U-test, chi-square, or Fischer's exact test. Significant differences (p < 0.05) are indicated by *. ISQ: Immune Status Questionnaire.

Variables	Total N=925	ISQ scores below cutoff value (N = 316) (34.17)	ISQ scores above cutoff value (N = 609) (65.83)	P
	Wight (kg)	68 (25)	69 (22)	65 (29)	0.006*
Height (m)	1.65 (0.15)	1.62 (0.15)	1.66 (0.15)	0.001*
BMI				
Underweight	93 (10.1)	48 (15.2)	45 (7.4)	0.001*
Normal	370 (40)	115 (36.4)	255 (41.9)	
Overweight	278 (30.)	79 (25)	199 (32.7)	
Obese	184 (19.9)	74 (23.4)	110 (18.1)	
Sleep (hours/night)	4 (3)	3 (3)	4 (2)	0.001*
>6	115 (12.4)	38 (12)	77 (12.6)	0.001*
4-6	382 (41.3)	105 (33.3)	277 (45.5)	
<4	428 (46.3)	173 (54.7)	255 (41.9)	
Smoking				
Yes	279 (30.2)	194 (61.4)	85 (14)	0.001*
No	600 (64.9)	101 (32)	499 (82)	
Ex-smoker	46 (5)	21 (6.6)	25 (4)	
Cigarette/day				0.07
1-10	123 (44)	93 (48.)	30 (35.3)	
11-20	81 (29)	53 (27.3)	28 (32.9)	
21-30	75 (27)	48 (24.7)	27 (31.8)	
Screen viewing time (hours/day)				
None	95 (10.3)	27 (8.5)	68 (11.2)	0.257
1-3 hours	286 (30.9)	106 (33.5)	180 (29.6)	
3-5 hours	258 (27.9)	80 (25.4)	178 (29.2)	
>5 hours	286 (30.9)	103 (33.6)	183 (30)	

**Figure 1:**
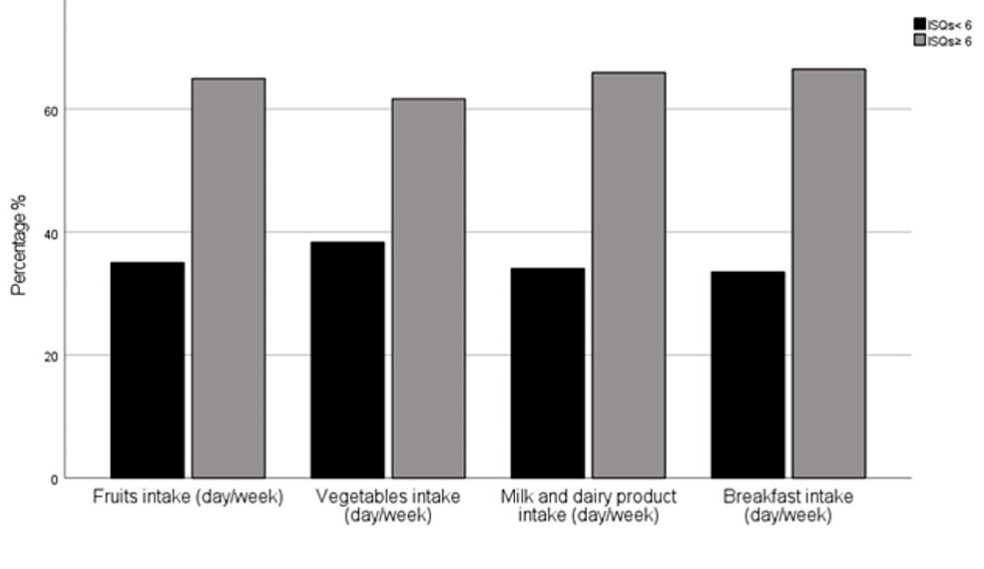
Proportion (%) of selected healthy dietary habits grouped by Immune Status Questionnaire (ISQ) scores among the enrolled study participants

A closer look at Figure 1 revealed that adherence to healthy dietary habits may improve immune health. Over 60% of the study participants with normal IS (ISQ ≥ 6) had breakfast, fruits, vegetables, or dairy products for three days or more each week, in contrast to their fellow participants, but without a statistically significant difference (P > 0.05).

Table 3 presents the physical activity profiles of the enrolled study participants stratified by the ISQ. It is apparent from this table that 60.2-82.7% of study participants were not engaged in any type of physical activity. No significant difference was evident between the various physical activities and IS groups, except for household activity (P = 0.02). Participants with normal IS spent significantly more time involved in such activity than those with an ISQ < 6 (P = 0.001).

**Table 3:** Physical activity profile of the enrolled study subjects grouped by ISQ scores Note: Data reported as mean (SD) or number (%). Test used = Mann-Whitney U-test, chi-square, or Fischer's exact test. Significant differences (p < 0.05) are indicated by *. ISQ: Immune Status Questionnaire.

Variables			Total (N = 925), N (%)	ISQ scores below cut-off value (N = 316) (34.17), N (%)	ISQ scores above cut-off value (N = 609) (65.83), N (%)	P-value
	No		313 (33.8)	102 (32.3)	211 (34.6)	0.259
Walking	Yes		612 (66.2)	214 (67.7)	398 (65.4)	
		Days/week	2.27 (2.2)	2.37 (2.22)	2.21 (2.18)	0.31
		15 minutes	95 (15.5)	43 (20.1)	52 (13.1)	0.070
		15-30 minutes	231 (37.7)	75 (35.0)	156 (39.2)	
		>30 minutes	286 (46.7)	96 (44.9)	190 (47.7)	
Jogging or running	No		563 (60.9)	186 (58.9)	377 (61.9)	0.204
	Yes		362 (39.1)	130 (41.1)	232 (38.1)	
		Days/week	1.21 (1.9)	1.29 (1.9)	1.17 (1.88)	0.21
		15 minutes	134 (39.5)	57 (43.8)	77 (33.2)	0.130
		15-30 minutes	117 (32.3)	38 (29.2)	79 (34)	
		>30 minutes	111 (30.7)	35 (27)	76 (32.8)	
Swimming	No		691 (74.7)	240 (75.9)	451( 74.1)	0.293
	Yes		234 (25.3)	76 (24.1)	158 (25.9)	
		Days/week	0.3 (0.95)	0.30 (1.06)	0.32 (0.9)	0.57
		15 minutes	107 (45.7)	43 (56.6)	64 (40.5)	0.066
		15-30 minutes	57 24.4)	14 (18.4)	43 (27.2)	
		>30 minutes	70 (29.9)	19 (25)	51 (32.3)	
Moderate‑intensity sports	No		627 (67.8)	210 (66.5)	417 (68.5)	0.291
	Yes		298 (32.2)	106 (33.5)	192 (31.5)	
		Days/week	0.57 (1.11)	0.54 (1.43)	0.62 (1.27)	0.60
		15 minutes	116 (38.9)	40 (37.7)	76 (39.6)	0.770
		15-30 minutes	83 (27.9)	28 (26.4)	55 (28.6)	
		>30 minutes	99 (33.2)	38 (35.8)	61 (31.8)	
Vigorous‑intensity sports	No		644 (69.6)	212 (67.1)	432 (70.9)	0.129
	Yes		281 (30.4)	104 (32.9)	177 (29.1)	
		Days/week	0.61 (1.4)	0.75 (1.62)	0.54 (1.26)	0.212
		15 minutes	101 (35.9)	32 (30.8)	69 (39.0)	0.380
		15-30 minutes	66 (23.5)	26 (25.0)	40 (22.6)	
		>30 minutes	114 (40.6)	46 (44.2)	68 (38.4)	
Self-defense	No		765 (82.7)	254 (80.4)	98 (16.1)	0.106
	Yes		160 (17.3)	62 (19.6)	511 (83.9)	
		Days/week	0.24 (0.96)	0.31 (1.09)	0.20 (0.87)	0.218
		15 minutes	93 (58.1)	32 (51.6)	61 (62.2)	0.379
		15-30 minutes	42 (26.3)	18 (29.0)	24 (24.5)	
		>30 minutes	25 (15.6)	12 (19.4)	13 (13.3)	
Resistant training	No		622 (67.2)	211 (66.8)	411 (67.5)	0.441
	Yes		303 (32.8)	105 (33.2)	198 (32.5)	
		Days/week	0.89 (1.7)	0.79 (1.6)	0.94 (1.7)	0.46
		15 minutes	122 (40.3)	36 (34.3)	86 (43.4)	0.280
		15-30 minutes	82 (27.1)	30 (28.6)	52 (26.3)	
		>30 minutes	99 (32.7)	39 (37.1)	60 (30.3)	
Household activity	No		416 (45.0)	127 (40.2)	289 (47.5)	0.02*
	Yes		509 (55.0)	189 (59.8)	320 (52.5)	
		Days/week	2.13 (2.5)	1.89 (2.4)	2.59 (2.66)	<0.001*

Table 4 provides an overview of the correlations between total ISQ scores and potential lifestyle variables that influence immune health status. A significant positive correlation was found with sex (P < 0.001), nationality (P = 0.011), weight (P = 0.003), height (P < 0.001), total sleep time per night (P < 0.001), and duration of household activity (P < 0.001). A significant negative correlation was observed between the number of cigarettes smoked per day (P = 0.035), fast food intake (P < 0.034), duration of vigorous or intense sports (P = 0.037), and resistance training (P = 0.037).

**Table 4:** Correlations of ISQ scores with different variables among the enrolled study participants Note: Test used = Spearman's or Pearson’s correlation coefficient (r). * P < 0.05, ** P < 0.001. ISQ: Immune Status Questionnaire.

Variable	r	P-value
Gender	0.158**	<0.001
Age (years)	-0.019	0.565
Nationality	0.084*	0.011
BMI (kg/m2)	-0.043	0.187
Wight (kg)	-0.099**	0.003
Height (m)	0.158**	<0.001
Sleep duration (hours/night)	0.130**	<0.001
Smoking cigarettes/day	-0.126*	0.035
Screen viewing time (hours/day)	-0.015	0.644
Fast food intake (>3 days/week)	-0.070*	0.034
Energy drinks intake (>3 days/week)	-0.009	0.793
Sweets/chocolate intake (>3 days/week)	-0.037	0.26
Sugar-sweetened drinks intake (>3 days/week)	-0.053	0.106
Household activity time (minutes)	0.101*	<0.001
Vigorous‑intensity sports time (minutes)	-0.065*	0.037
Resistance training duration time (minutes)	-0.103*	0.037
Perceived health	0.313**	<0.001
Perceived immune functioning	0.212**	<0.001

## Discussion

In recent years, Saudi society has undergone substantial lifestyle changes that have resulted in the acquisition of potentially harmful lifestyle habits, which pose a risk to long-term health as well as immune health and may result in an increased risk of disease, the treatment of which can place a significant burden on the healthcare system [29]. To the best of our knowledge, this is the first study to use the ISQ to assess the status of immune health among residents of Saudi Arabia's second-largest province, the Western Province, and to investigate its association with commonly adopted lifestyle habits that are thought to have an impact on immune functions. Few studies, both locally and globally, have reported the immune status of healthy adults. One-third of the 925 participants in the current study had a low immune status score, as defined by an ISQ score < 6. These values exceed the 17.5% previously recorded by another Saudi Arabian study [9] and are compatible with the findings of a study of young Dutch adults [16]. Nonetheless, they are lower than the United States' numbers [30]. This may be due to the increased incidence of obesity and its related risk factors in the US population [31,32]. The fact that more than half of our group sample was overweight or obese demonstrates the prevalence of unhealthy lifestyle choices. Of the participants, 30% spent more than five hours a day viewing screens. In addition, 46.3% of the participants slept less than four hours per night. Finally, 30.2% of the participants were active smokers. The high prevalence of these behaviors among young, healthy people may contribute to a low ISQ score. Our findings are consistent with those of previous research on the effect of obesity on immunological fitness, which demonstrated a correlation between weight and ISQ scores. Obesity is a distinct risk factor [33]. Obesity has recently been associated with higher mortality and disease severity during the COVID-19 pandemic [10]. To boost immunological health and prepare for potential infectious pandemics, it was recently suggested that healthy body weight be maintained [11]. Evidence links immune system dysfunction to several different types of sleep disorders, such as circadian rhythm disruption and sleep apnea [16]. Another study found that a lack of quality sleep was linked to lowered immunity and a longer time for wounds to heal [34]. Other studies have found that getting too little sleep (less than five hours each night) can negatively affect the immune system [19]. Smoking affects the immune system, making it more susceptible to illness, and has a particularly negative impact on macrophage and cytokine responses, making it more difficult to fight infection. In our group, smoking status was significantly correlated with immune health status [35]. In terms of physical activity, our data show that the most common activity was related to household tasks. Despite its being a low-intensity activity, it has been shown to have a considerable effect on immunity. The well-known benefits of physical activity notwithstanding, many participants (60-80%) did not engage in any type of physical activity. This could explain why physical activity had no effect on the immunological state in our study. When it comes to dietary habits and their impact on good immune health status as defined by ISQ score ≥ 6, the findings revealed a positive relationship between participants' immune status and their intake of healthy foods, even if this association was not statistically significant. The correlation analysis consistently indicated a potential negative contribution of fast food intake (> three days/week) to total ISQ. This finding broadly supports those of other studies in this field that link a healthier diet to immune fitness and immune biomarkers [12]. It is somewhat surprising that a weak correlation between the total ISQ score and other dietary habits, such as energy drink consumption, sugar-sweetened beverage consumption, and sweet or chocolate consumption, was noted in this condition. This could be due to a social desirability bias, as participants were more likely to report positive behaviors. In the present study, sociodemographic characteristics, including gender differences, residential location, and nationality variance, showed statistical significance among residents of the Western Province, along with the residential location. These results are in agreement with Melaku et al.’s (2019) finding, which showed that the most significant predictors of immune fitness status and mortality from all causes were identified as socioeconomic variables. These findings emphasize the necessity of addressing social inequalities and reinforcing behavioral interventions for various segments of the population [36].

The current investigation had a few drawbacks due to the fact that lifestyle risk factors were evaluated by self-administered questionnaires. Social desirability bias can result in non-random misclassification, as individuals are more likely to report positive behaviors. This means that the indicators of healthy living, including exercise and diet, were probably underestimated. Moreover, selection bias may have been present in the convenience sample as well as the online self-administered questionnaire. Finally, it is possible that the findings of this study are applicable to only one region in Saudi Arabia.

## Conclusions

The survey data presented here show a link between immune health and lifestyle among residents of Saudi Arabia's Western Province. We demonstrated that a variety of harmful lifestyle habits are widespread and may have a negative impact on the public's immune health. It also emphasizes the critical need to implement various measures to mitigate the negative impact of an unhealthy lifestyle on public health and to reverse the effects of poor immune health that were observed.
